# Fermented pork fat (Sa-um) and lifestyle risk factors as potential indicators for type 2 diabetes among the Mizo population, Northeast India

**DOI:** 10.1186/s41043-021-00257-8

**Published:** 2021-07-22

**Authors:** Freda Lalrohlui, Souvik Ghatak, John Zohmingthanga, Vanlal Hruaii, Nachimuthu Senthil Kumar

**Affiliations:** 1 Department of Biotechnology, Mizoram University, Aizawl, Mizoram 796004 India; 2 Department of Pathology, Civil Hospital, Aizawl, Mizoram 796001 India; 3 Department of Medicine, Zoram Medical College, Aizawl, Mizoram 796005 India

**Keywords:** Mizo population, Type 2 diabetes mellitus, Dietary habits, Sa-um, Tuibur, Biochemical profiles

## Abstract

Over the last few decades, Mizoram has shown an increase in cases of type 2 diabetes mellitus; however, no in-depth scientific records are available to understand the occurrence of the disease. In this study, 500 patients and 500 healthy controls were recruited to understand the possible influence of their dietary and lifestyle habits in relation with type 2 diabetes mellitus. A multivariate analysis using Cox regression was carried out to find the influence of dietary and lifestyle factors, and an unpaired *t* test was performed to find the difference in the levels of biochemical tests. Out of 500 diabetic patients, 261 (52.3%) were males and 239 (47.7%) were females, and among the control group, 238 (47.7%) were males and 262 (52.3%) were females. Fermented pork fat, Sa-um (odds ratio (OR) 18.98), was observed to be a potential risk factor along with tuibur (OR 0.1243) for both males and females. Creatinine level was found to be differentially regulated between the male and female diabetic patients. This is the first report of fermented pork fat and tobacco (in a water form) to be the risk factors for diabetes. The unique traditional foods like Sa-um and local lifestyle habits like tuibur of the Mizo population may trigger the risk for the prevalence of the disease, and this may serve as a model to study other populations with similar traditional practices.

## Introduction

Type 2 diabetes mellitus (T2DM) is a polygenic disorder which arises mainly when the body cannot utilize insulin effectively. The pancreas secretes insulin hormone that helps to carry glucose from the bloodstream into the cells [1]. Elevation in the blood sugar level may lead to acute and long-term complications. T2DM is a chronic illness that requires self-management and medical care which proves to be a complex issue [2]. Diabetes mellitus has been purposively chosen for analysis on the ground that it still constitutes one of the major public health problems worldwide. Around the world, the majority of people with diabetes consists mainly of type 2 diabetes, which is found to be the result of physical inactivity and excess body weight. About 2.2 million deaths in 2012 and 1.6 million deaths in 2015 were observed due to the direct cause of diabetes [3]. According to the International Diabetic Federation (IDF), the prevalence of diabetes constitutes 8.7% of the population in Southeast Asia where 51.1% are undiagnosed. In India, about 63 million people had diabetes in 2011 with the age range of 20 to 79 years and are expected to rise by 101 million by 2030 [4]. The lifestyle changes, habitual practices, or unique prolonged habits may lead to an increase in the incidence of T2DM, and these factors may also vary among the ethnic groups [5].

The risk of T2DM may be triggered by various dietary factors which may include consumption of a high-calorie diet, beverages, and refined foods [6]. As people get older, the risk of T2DM may also increase, especially after the age of 45 years which may be due to less exercise, loose muscle mass, and increase in body weight [7]. Modernization and westernization of lifestyle and dietary habits in developing countries revealed many changes towards a certain type of genes, which may have an adverse effect genetically and in turn lead to susceptibility to type 2 diabetes in different ethnic groups. People became less involved in physical activity while the dietary intake and consumption of meat increases which are consistently associated with risk for diabetes [8, 9].

In a small tribal state like Mizoram, the disease occurrence has been reported to be increasing over time, and on contrary, records found in the health centers, clinics, and hospitals are fragmentary, discontinuous, and incomplete to make meaningful dis-aggregation at lower levels and it is difficult to probe the frequency of occurrence [5]. Mizoram is a hilly mountainous region of the Eastern Himalayas and belongs to one of the eight sister states of the North-eastern region of India, bordered by Bangladesh and Myanmar, and is the second least populous state in the country [10]. Tobacco in different forms is used more frequently and a peculiar habit of consumption of tobacco smoke-infused aqueous water (“tuibur”) by the Mizo-Mongloid tribe [11, 12]. The people of Mizoram have the habit of chewing “kuhva” (betel leaf with raw areca nut and excess lime) and consumption of smoked meat or vegetables. Rice is the staple food for Mizo tribal people and is mostly favored for breakfast and dinner, while any other foods such as vegetables and meats are considered as side dishes. Spices tend to be used less when compared with other Indian cuisines [13]. Different types of fresh leafy vegetables such as mustard (antam) and pumpkin (maian) leaves are served, and food varieties such as bamboo shoot (mautuai, rawtuai), fermented lard (Sa-um), fermented soya beans (bekang), and dried fish are preserved through smoking. The foods are mostly prepared and served as boiled, stewed, steamed, smoked, or fermented form. Slight variation and modifications in the processing techniques may occur from place to place for ease of preparation and for quality improvement [14].

These unique lifestyle factors of the Mizo people might play a role in the high incidence of T2DM, and the present study was carried out to understand the influence of demographic and epidemiological factors that may contribute to the risk. The study also focuses on the biochemical profiles and their gender-specific variation in type 2 diabetes.

## Materials and method

### Participants

Five hundred previously diagnosed T2DM patients receiving care from major diagnostic and health clinic “Genesis Laboratory,” Aizawl, Mizoram, and five hundred controls volunteered to participate with consent in this study during January 2016–December 2017. The age range for individuals with T2DM and healthy controls was between 40 and 85 years (mean age range = 65.5 years) were included in this case-control study. The diabetic patients were all previously diagnosed based on the WHO criteria [15]. The work has been approved by ethical committees of Civil Hospital, Aizawl (B.12018/1/13-CH(A)IEC/39 dtd. 23/12/2015), and Human Ethical Committee, Mizoram University (MZU/IHEC/2015/006 dtd. 14/12/2015).

Inclusion criteria for this study included the selection of individuals with T2DM after confirmation by a diabetologist, fasting plasma glucose levels > 120 mg/dl, and post-prandial plasma glucose levels > 200 mg/dl. The healthy control group comprised of non-diabetic subjects without the symptoms of diabetes mellitus and blood glucose levels within normal limits (below < 120 mg/dl). The individuals with T2DM and healthy controls included were from the Mizo ethnic group. The exclusion criteria for sampling included individuals with T2DM with gestational diabetes, diabetes due to pancreatic disorder, pregnancy, and other types of severe illness and metabolic diseases.

### Study design and assessment

The epidemiological information for this case-control study was collected with the consent of the participants. Information on demographic factors, dietary habits, tobacco, alcohol use, and family history was recorded. Dietary habits like meat, smoked meat, salt intake, and Sa-um were taken into account. Lifestyle habits such as cigarette, sahdah, tuibur, paan, and alcohol consumption were recorded (Table 1). Dietary habits were grouped into three categories, viz., low (1 to 2 days per week), moderate (2 to 3 days per week), and high (more than 3 days per week). Blood sugar was estimated by the glucose oxidase-peroxidase (GOD-POD) method, and cholesterol level was estimated by the enzymatic method using cholesterol oxidase/peroxidase aminophenazone (CHOD-PAP reagent) using Sys200 Biochemistry Analyzer [16–18]. Estimation of creatinine was done by the modified Jaffe’s method by using a Sys 200 biochemistry analyzer [16, 19]. The type of medication taken by the patients was recorded as oral or injection. The history of hypertension was also taken into account. Body mass index (BMI) was calculated as weight (kg)/height^2^ (m^2^) and used to categorize BMI-measured weight status: underweight (BMI ≤ 18.5), normal (BMI 18.5–24.9), overweight (BMI 25.0–29.9), and obese (BMI ≥ 30) [20, 21].

**Table 1:** Significant lifestyle and dietary factors used in the univariate analysis. The univariate analysis was carried out to rule out the factors that are deemed to be important to be further analyzed using the multivariate analysis. The odds ratio between the diabetic patients and healthy controls was compared. The 95% CI value was obtained between the cases and the control with a significant value of *p* (< 0.05)

Factors	Odds ratio	95% CI	***p*** value
Gender	1.024	0.799–1.313	0.8493
Age (> 45 diabetic vs > 45 years control )	0.0893	0.0426–0.1871	< 0.0001
Education (diabetic vs control)	1.113	0.833–2.141	1.4235
Income (diabetic vs control)	1.089	0.801–1.475	0.9578
Cigarette consumption	0.7465	0.5940–0.9382	< 0.0122
Alcohol consumption	0.0117	0.0016–0.0844	< 0.0001
Paan consumption	0.0571	0.0348–0.0939	< 0.0001
Sahdah consumption	1.8776	1.5139–2.3286	< 0.0001
Tuibur consumption	2.0130	1.3908–2.9137	0.0002
Sa-um consumption	2.2667	1.7115–3.0019	< 0.0001
Meat consumption	0.4115	0.3344–0.5064	< 0.0001
Smoked meat consumption	0.0695	0.0499–0.0970	< 0.0001
Salt consumption	0.2882	0.2309–0.3597	< 0.0001

### Statistical analysis

The various lifestyle and dietary factors were analyzed between individuals with T2DM and healthy controls. The statistical analysis was performed using SPSS 20.0 version (IBM Corp, Armonk, NY) software package. A chi-square test was used to assess the association between demographic factors and type 2 diabetes. A logistic regression analysis was carried out to calculate the influence of lifestyle and dietary factors for T2DM. Factors that were deemed of potential importance in the univariate analysis (*p* < 0.05) were further analyzed in the multivariate analysis using Cox regression [22]. The independent effect of risk factors was investigated in a multivariate model (introducing all variables and terms of interactions) retaining the statistically significant factors which show a confounding effect only. Gender and various lifestyle and food habits were all considered in the regression model as potential confounders to evaluate their association with T2DM. The variables were adjusted with age. A receiver operating characteristic (ROC) curve was plotted to find the specificity and sensitivity for the risk factors which achieved a high odds ratio and also to estimate their potential risk score. An unpaired *t* test was performed for male and female diabetic patients to find the difference in the levels of biochemical tests.

## Results

**Fig. 1:**
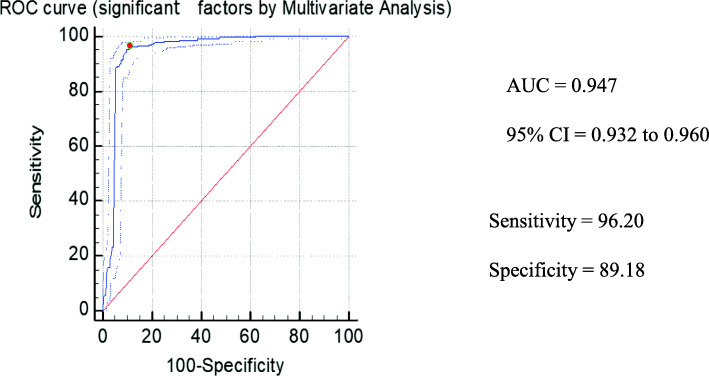
Estimated ROC curve for significant demographic factors from the multivariate analysis

**Fig. 2:**
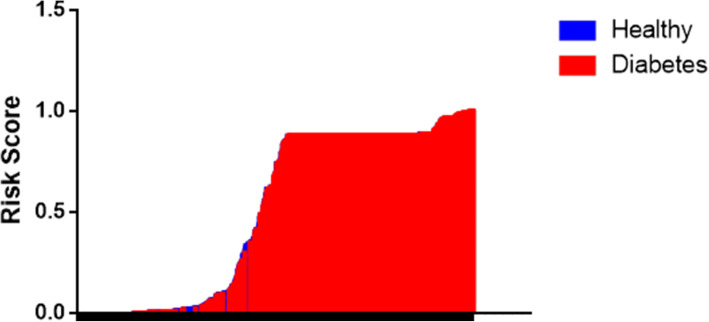
Estimated risk for the significant demographic factors between diabetic and healthy controls using the multivariate analysis

The total number of participants was 1000 (500 diabetic and 500 controls). Out of 500 diabetic patients, 261 (52.3%) were males and 239 (47.7%) were females, whereas among control groups, 238 (47.7%) were males and 262 (52.3%) were females. From the results of the univariate analysis (Table 1) using the chi-square test, the factors that were considered to be of potential importance like alcohol, paan, sahdah, tuibur, Sa-um, meat, smoked meat, and salt were further analyzed using the multivariate analysis. The risk of type 2 diabetes was higher in patients who consumed Sa-um which is a fermented pork fat (odds ratio (OR) 18.98, 95% confidence interval (95% CI) 9.8182–36.6918). This was observed with the adjusted age of 45 years or more for both males and females (OR 0.0893, 95% CI 0.0419–0.5300). Other lifestyle risk factors include betel leaves with areca nut chewing (OR 0.1006, 95% CI 0.0537–0.1885), tuibur (OR 0.1243, 95% CI 0.0530–0.2918), and dietary habits like smoked meat (OR 0.0703, 95% CI 0.0412–0.1200). Consumption of salt in excess may also be a risk for type 2 diabetes (OR 0.2134, 95% CI 0.1400–0.3251) (Table 2). The area under the ROC curve is 0.947 which is predicted to be a potential hazard score for these factors (Figs. 1 and 2). There was no correlation found between BMI and type 2 diabetes in the Mizo population.

**Table 2:** Significant lifestyle and dietary factors used in the multivariate analysis. The factors that were deemed to be of potential significance from the univariate analysis were further analyzed using the multivariate analysis. The odds ratio between the diabetic patients and healthy controls was compared. The 95% CI value was obtained between the cases and the control with a significant value of *p* (< 0.05)

Factors	Odds ratio	95% CI	***p*** value
**Age in years (> 45 diabetic vs > 45 control)**	0.1491	0.0419–0.5300	0.0033
**Paan consumption**	0.1006	0.0537–0.1885	< 0.0001
**Tuibur consumption**	0.1243	0.0530–0.2918	< 0.0001
**Sa-um consumption**	18.9802	9.8182–36.6918	< 0.0001
**Smoked meat consumption**	0.0703	0.0412–0.1200	< 0.0001
**Salt consumption**	0.2134	0.1400–0.3251	< 0.0001

**Fig. 3:**
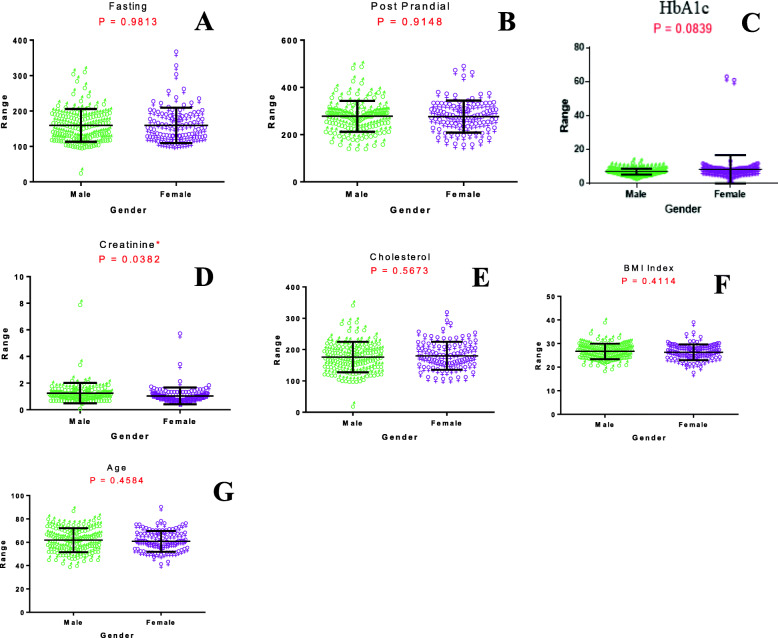
Scatter plot showing the comparison of different clinical factors (after performing an unpaired *t* test) between male and female diabetic patients. Comparison of **A** fasting blood sugar, **B** post-prandial blood sugar, **C** HbA1c, **D** creatinine, **E** cholesterol, **F** BMI index, and **G** age

Between the male and female diabetic patients, we observed no difference in biochemical parameters like fasting glucose level (*p* = 0.9813), post-prandial glucose level (*p* = 0.9148), cholesterol (*p* = 0.5673), and HbA1c (*p* = 0.0839). However, creatinine level (*p* = 0.0382) was observed to be differentially regulated between male and female diabetic patients in the Mizo population (Fig. 3).

## Discussion

The objective of the study focuses solely on the role of demographic factors which may contribute to the onset of diabetes in the Mizo population. Although the prevalence of T2D increases with obesity, old age, and family history of diabetes, there is also the involvement of certain genetic factors as well as demographic factors [23]. From our study, we found that the Mizo population has their own unique lifestyle and dietary habits which may attribute to the prevalence of type 2 diabetes in Mizoram. The risk of type 2 diabetes was found to be higher in patients consuming Sa-um which is an animal fat product with caul fat adipose tissue which is prepared semi-dry in bottle gourd (*Lagenaria siceraria*) and although it exhibits distinct astringency it has no significant organoleptic qualities. Sa-um which is also a derivative of pork fat is one of the traditional foods for the local people although it exemplifies adverse health characteristics due to the presence of high saturated fat/cholesterol content [24, 25]. In contribution to Sa-um, smoked meat and excess salt consumption may act as secondary risk factor for type 2 diabetes in Mizoram. Mizo people still practice a variety of food processing habits that were passed on from their forefathers. Only the smoke and the heat directly affect the meat in the traditional smoking process and the meat is generally not barbecued. High-heat cooking many produce harmful chemicals such as polycyclic aromatic hydrocarbons, heterocyclic aromatic amines, and nitrosamines (from nitrates and nitrites added to meats as a preservative) which may lead to an inflammatory response and interfere with the normal production of insulin [26]. High salt or excess salt intake may lead to insulin resistance which in turn may lead to hyperglycemia. Lifestyle factors like betel nut chewing and tuibur consumption also attribute to the risk of developing type 2 diabetes. Some studies show that betel nut chewers may develop T2DM at an early stage which may peak up later in life around the age of 60–69 years [27]. Tuibur is a tobacco-infused water and tobacco is known to be linked with many diseases and nicotine’s ability to affect certain antioxidant enzymes like lipid peroxidase, superoxide dismutase, etc., which in turn may contribute to the development of T2DM [28]. Studies from different populations reported that smoking has an impact on T2DM development [29], as chronic smokers have a higher risk of insulin resistivity [30,31, 32, 33]. Since many risk factors especially those which are modifiable undergo certain changes which may be unfavorable and can in turn cause prevalence of T2DM [34]. However, in our study, we observed no significant relationship between smoking and T2DM in this population [30]. The levels of creatinine may also play a crucial role in depicting the risk for T2DM, especially lower creatinine level, as it is the only substance that is produced by the skeletal muscle mass, which in turn may have a regulatory effect with glucose uptake, thus leading to insulin resistance [35, 36]. Some of the risk factors that can cause fluctuation in the creatinine levels are cigarette smoking, larger body weight and height, male gender, and old age [37]. Since creatinine level corresponds to muscle mass of the body, the level becomes very likely to increase in males than in females [38,39].

The strength of the present study may include the in-depth interview with the participants, large study samples in spite of the small tribal population, standardized measurement for the biochemical profiles in a single laboratory, and adjustment for several confounding factors like meat consumption, alcohol consumption, and family history as well as population-based habits like sahdah (dried tobacco leaves) chewing which were all taken into consideration. To our knowledge, the present study is the first approach to understand the possible relations between unique dietary and lifestyle habits and type 2 diabetes in a tribal population. Although modernization plays an active role in the diet and lifestyle of the people, still diets like Sa-um and smoked food and habits like kuhva and tuibur consumption are still practiced, which may trigger the risk for type 2 diabetes. Thus, the food and lifestyle habits practiced by the people can influence the risk of various diseases, leading to a gradual increase in the acceleration of the disease. A further in-depth study on T2DM involving more samples as well as consumption of various junk foods as per the modern lifestyle habits is required to fully understand the incidence of the disease.

## Data Availability

Please contact the authors for data requests.
